# A novel cross‐species *p*‐tau217 immunoassay for investigating animal models of Alzheimer's disease

**DOI:** 10.1002/alz70856_104853

**Published:** 2026-01-07

**Authors:** Alberto González‐Mayoral, Fernando Gonzalez‐Ortiz, Daniella Balduino Victorino, Marie‐Claude Potier, Kaj Blennow, Nicolas Villain

**Affiliations:** ^1^ Sorbonne Université, INSERM U1127, CNRS 7225, Paris Brain Institute ‐ PBI, Paris, France; ^2^ University of Gothenburg, Department of Psychiatry And Neurochemistry, Institute of Neuroscience And Physiology, The Sahlgrenska Academy At The University Of Gothenburg, Mölndal, Sweden; ^3^ Sahlgrenska University Hospital, Clinical Neurochemistry Laboratory, Mölndal, Sweden; ^4^ Département de Neurologie, Groupe Hospitalier Pitié‐Salpêtrière, AP‐HP Sorbonne Université, Institute of Memory and Alzheimer's Disease, Paris, France; ^5^ Sorbonne Université, INSERM U1127, CNRS 7225, Institut du Cerveau ‐ ICM, Maladie d’Alzheimer, Maladies à Prions, Paris, France, Paris, France

## Abstract

**Background:**

The measurement of phosphorylated tau (pTau) in biofluids, particularly at position 217 (pTau217), has proven to be a valuable diagnostic and prognostic marker in Alzheimer's disease (AD). However, due to tau variability across species existing immunoassays optimized for human lack compatibility with mouse models, posing a challenge for studying these markers in preclinical research. Single Molecule Array (Simoa) technology offers unparalleled sensitivity and precision, making it an ideal platform for developing novel assays to address these challenges.

**Methods:**

Human and mouse tau isoforms were analyzed via sequence alignment to identify conserved regions suitable for cross‐species antibody targeting.

Western blot (WB) and Enzyme‐Linked Immuno Sorbent Assay (ELISA) experiments evaluated cross‐reactivity of human tau antibodies with mouse tau on brain extracts.

A novel pTau217 immunoassay was developed and validated on the Simoa platform with plasma samples from three distinct mouse models containing either endogenous or transgenic human tau (APP23, P301S and WT) and human plasma samples for cross‐species validation. Optimization for sensitivity, specificity, and reproducibility included different antibodies concentration tests, limit of quantification, and buffer adjustments to diminish plasma matrix interference.

**Results:**

The sequence alignment, WB and ELISA analyses guided the selection of antibodies targeting conserved epitopes, ensuring cross‐species applicability. The novel pTau217 immunoassay, based on Simoa technology, demonstrated good performance in detecting pTau in brain extracts and plasma across all three mouse models containing endogenous mouse tau and transgenic human tau. It also proved to detect human plasma pTau217. The assay exhibited high sensitivity, achieving reliable detection of pTau even in plasma samples with low abundant protein concentrations and matrix interference effect, and showed robust specificity to pTau217.

**Conclusions:**

We present the validation of the first Simoa‐based pTau217 immunoassay designed to measure *p*‐tau across species (mouse and human), whether in plasma samples with genuine mouse tau sequence or transgenic human tau sequence. Further development will focus on linearity tests and correlation with neuropathologic changes. Our findings have broad implications for biomarkers research and help bridge the gap between human and animal studies in AD research.

